# Sex-specific aspects in the development of tissue metabolic damage in a non-obese prediabetic model

**DOI:** 10.3389/fendo.2026.1786893

**Published:** 2026-04-14

**Authors:** Martina Hüttl, Irena Markova, Iveta Zapletalova, Hana Malinska

**Affiliations:** 1 Center for Experimental Medicine, Institute for Clinical and Experimental Medicine, Prague, Czechia; 2 Department of Pharmacology, Faculty of Medicine and Dentistry, Palacky University, Olomouc, Czechia

**Keywords:** hypertriglyceridemia, inflammation, insulin resistance, lipid metabolism, NAFLD, prediabetes, sex differences

## Abstract

**Background:**

Recent studies suggest that the development of prediabetes and its associated comorbidities may depend on sex and reproductive status. While the exact mechanism is unclear, differences in insulin sensitivity, body fat distribution, and glucose and lipid metabolism may play a role. In this study, we investigated how sex differences in metabolic and inflammatory parameters affect the development of prediabetic conditions in a non-obese rat model with severe dyslipidaemia.

**Methods:**

Wistar Kyoto (WKY) rats served as the control group, while age-matched Hereditary Hypertriglyceridaemic (HHTg) rats were used as a non-obese, prediabetic model with genetically determined hypertriglyceridaemia, insulin resistance and impaired glucose tolerance.

**Results:**

Compared to WKY controls, the HHTg strain exhibited increased serum triacylglyceroles (TAG) as well as ectopic TAG accumulation in the liver, heart and skeletal muscle which was more pronounced in HHTg females. However, this higher ectopic TAG accumulation in HHTg females was not associated with increased lipotoxic diacylglyceroles. The HHTg strain showed increased visceral adiposity, which was distributed differently: HHTg females had increased perimetrial adipose tissue, while HHTg males had increased perirenal adipose tissue. Impaired insulin sensitivity was observed in both sexes of the HHTg strain in skeletal muscle and adipose tissue. Insulin resistance in the HHTg strain may be due to elevated leptin and NEFA levels, as well as decreased GLUT4 in skeletal muscle. In addition, the HHTg strain showed impaired glucose tolerance, as well as hyperinsulinaemia, which was more pronounced in HHTg males. Increased lipogenesis (*mRNA Scd1*), oxidative stress (decreased SOD activity) and inflammation (*mRNA Tnfα) in the liver may contribute to the development of hepatic steatosis and hepatic lipid accumulation. In visceral adipose tissue, increased mRNA Hif1* may contribute to adipose tissue hypoxia and impair insulin sensitivity, particularly in males.

**Conclusions:**

Despite having more pronounced dyslipidaemia, ectopic lipid accumulation, and visceral adiposity, prediabetic females have better glucose tolerance and insulin sensitivity markers than prediabetic males. These sex differences may be due to variations in fat distribution, lipid metabolism and chronic inflammation. Our findings suggest that males are more susceptible to developing early prediabetic damage, such as insulin resistance and fatty liver, regardless of obesity.

## 1 Introduction

The prediabetic state and metabolic syndrome are associated with a number of metabolic disorders, with insulin resistance (IR) and impaired lipid metabolism being early manifestations. Dyslipidaemia, particularly hypertriglyceridaemia, is a characteristic of the prediabetic condition and is followed by ectopic lipid deposition. This plays an important role in tissue impairment, including IR, chronic inflammation and oxidative stress (1). Alongside impaired lipid metabolism and ectopic lipid deposition, dyslipidaemia is one of the most common disorders associated with prediabetic conditions and often precedes the onset of hyperglycaemia. Furthermore, genetic background, lifestyle as well as sex and reproductive status can influence the development of prediabetes and its metabolic conditions (2). Sex-specific differences in the development of prediabetic impacts can also affect mortality rates and diagnostic and treatment strategies (3).

Recent epidemiological data suggest that men are at a higher risk of developing diabetes than women, which is likely to be related to their higher prevalence of IR (4). Despite having a higher BMI, women have higher fasting glucose and are less insulin resistant than men. Furthermore, the improved insulin sensitivity observed in women only occurs when glucose levels are normal and gradually disappearing in cases of hyperglycaemia and type 2 diabetes (5), as well as after menopause (6).

Sex differences are evident from the beginning of development and during prediabetic states. These differences may be due to variations in insulin sensitivity, body fat composition, and lipid metabolism. However, the underlying mechanisms are not well understood. The direct effect of sex hormones, as well as other mechanisms, is involved in this process. It is crucial to understand sex-specific differences in metabolic disorders associated with prediabetes and diabetes in order to develop personalized and precise treatment strategies for individuals at different stages of glucometabolic derangement.

Animal studies also suggest that there are sex-related differences in the development of metabolic disorders such as IR and glucose intolerance. In rodent models of IR and obesity, it is male rather than female animals that develop obesity-induced IR and glucose intolerance when fed a high-fat diet (7, 8). Most experimental research into sexual dimorphism has focused solely on diet-induced IR and obesity. These studies have suggested distinct responses to a high-fat diet in male and female mice (9, 10).

In this study, we investigated sex differences in metabolic and inflammatory parameters in relation to the development of prediabetic conditions using a non-obese prediabetic model exhibiting severe dyslipidaemia and IR, the Hereditary Hypertriglyceridaemic (HHTg) rats (11, 12). Severe dyslipidemia in this rat strain was exhibited by genetically determined hypertriglyceridaemia that is strongly associated with IR and the prediabetic condition. The present study followed up on our previous results (13, 14), revealed that HHTg female rats showed milder hypertension and less pronounced inflammation in the visceral adipose tissue and the aorta than HHTg males. Based on our previous results, female prediabetic rats are better protect from vascular dysfunction than prediabetic males despite exhibiting more pronounced dyslipidemia and pro-coagulation status (13).

## 2 Methods

### 2.1 Animals and experimental procedure

All experiments were performed in agreement with the Animal Protection Law of the Czech Republic (359/2012) and the Directive 2010/63/EU of the European Parliament and of the Council, and were approved by the Ethics Committee of the Institute for Clinical and Experimental Medicine (protocol number 35/2022). The study was performed on 6-month-old male (n=8) and female (n=8) Wistar Kyoto (WKY) rats as the control group and 6-month-old male (n=8) and female (n=8) HHTg rats (provided by the Institute for Clinical and Experimental Medicine, Prague, Czech Republic) as the non-obese prediabetic model. This rat strain exhibits genetically determined hypertriglyceridaemia, insulin resistance in peripheral tissues and liver steatosis but with absence of obesity, fasting hyperglycaemia, or essential hypertension. Rats were held under temperature- (22 °C) and humidity-controlled conditions under a 12-h/12-h light/dark cycle with free access to food (maintenance diet for rats and mice; Altromin, Germany) and drinking water. Male and females of WKY and HHTg rats were randomized into experimental groups. At the end of the experiment, the rats were decapitated in a postprandial state after being given a light anesthesia (zoletil 5mg/kg of body weight, intraperitoneally). Aliquots of serum and tissue samples were collected and stored at -80 °C for subsequent analysis.

### 2.2 Analytical methods and biochemical analysis

Serum levels of triacylglyceroles (TAG), glucose, non-esterified fatty acid (NEFA), total and HDL cholesterol were measured using commercially available kits (Erba Lachema, Brno, Czech Republic and Roche Diagnostics, Mannheim, Germany).

Serum insulin, glucagon, leptin and ghrelin concentrations were determined using the Rat ELISA kit (Mercodia AB, Sweden; Alpha Diagnostics International, USA; Biovendor, Czech Republic; Millipore, USA, respectively). The enzymatic spectrophotometrical method (available kit from Roche Diagnostics, Germany) was used to determine serum uric acid.

For the oral glucose tolerance test (oGTT), blood glucose levels were determined after a an intragastric glucose load (300 mg/100g of the body weight) administered intragastrically after overnight fasting. Blood was drawn from the tail before the glucose load at 0 min and 30, 60, 120 and 180 min thereafter. HOMA-IR (homeostatic model assessment for insulin resistance) was calculated as fasting insulin*138*fasting glucose/22.5.

The serum levels of 17β-estradiol were determined using an ultra-sensitive radioimmunoassay kit (Immunotech, Prague, Czech Republic). Serum testosterone was analyzed by rat ELISA kit (Crystal Chem, Elk Grove Village, IL, USA).

To determine the levels of triacylglyceroles (TAG) and cholesterol in the tissues, the samples were extracted using chloroform and methanol. The resulting pellet was dissolved in isopropyl alcohol, and the triacylglyceroles or cholesterol content was then determined by enzymatic assay (Erba-Lachema, Czech Republic) (15). To determine the content of diacylglyceroles (DAG) in the liver and skeletal muscles, the samples were extracted in dichloromethane/methanol. The resulting pellet was dissolved in isopropyl alcohol and isolated by thin-layer chromatography. The content of the individual lipid classes was then determined using an enzymatic assay (Erba-Lachema, Czech Republic; Roche Diagnostics, Germany).

Activity of antioxidant enzymes superoxide dismutase (SOD) and glutathione peroxidase (GPx) in the liver were analyzed using the Cayman Chemicals assay kits (Ann Arbor MI, USA).

### 2.3 Insulin sensitivity parameters in tissues

To measure the basal and insulin-stimulated incorporation of glucose into lipids or glycogen, epididymal adipose tissue or skeletal muscle (diaphragma) was incubated for 2 hours in Krebs-Ringer bicarbonate buffer, pH 7.4, containing 0.1 μCi/ml of ^14^C-U glucose, 5 mmol/L of unlabeled glucose, and 2.5 mg/ml of bovine serum albumin (Fraction V, Sigma, Czech Republic), with or without 250 μU/ml insulin. Lipid or glycogen extraction was followed by the determination of basal and insulin-stimulated glucose incorporation into lipids or glycogen (15). The protein content of skeletal muscle and epididymal or perimetrial adipose tissue was analyzed using the Lowry method (16).

### 2.4 Fatty acid composition and fatty acid desaturase activity

To determine the fatty acid composition in the liver, the extraction, separation and methylation of phospholipids were performed as previously described (15). Briefly, total lipids in the liver samples were extracted in dichloromethane/methanol using a modified Folch method. The organic phase was evaporated under N_2_ and the resulting pellet dissolved in an isopropyl alcohol/hexane mixture. The phospholipid classes were separated by thin-layer chromatography using hexane-diethyl ether-acetic acid (70:30:1, *v/v*) as a solvent system, extracted from silica gel, and then converted to fatty acid methyl esters (FAME). The fatty acid in phospholipid class was established by gas chromatography using the Hewlett-Packard GC system with hydrogen as the carrying gas, a flame ionization detector, and a carbowax-fused silica capillary column (Varian, USA). Individual FAME peaks were identified by comparing retention times with those of authentic standards (a mixture of fatty acids, Restek, USA) and the fatty acid composition was reported as a percentage of the total fatty acids (17).

### 2.5 Relative mRNA expression

Total RNA was isolated from tissues using RNA Blue (Top-bio, Czech Republic). Reverse transcription and quantitative real-time PCR analyses were performed using the TaqMan RNA-to C_T_ 1-Step Kit and TaqMan Gene Expression Assay (Applied Biosystems, USA) and carried out using a ViiA™ 7 Real Time PCR System (Applied Biosystems, USA). Relative expression was determined by normalizing against *Hprt1* as an internal reference, and was calculated using the 2^-ΔΔCt^ method. The results were run in triplicate.

### 2.6 Statistical analysis

Statistical analysis was performed using GraphPad Prism 11 (GraphPad Software, USA). Sample size was determined based on expected effect sizes derived from published comparisons of HHTg and WKY rats, in which metabolic phenotype differences yield large effects (Cohen’s f>0.65). *A priori* power analysis (α=0.05, power=0.80) confirmed that n=8 per group was sufficient to detect effects of this magnitude. Power analysis was done using R version 4.5.0. A two-way ANOVA was used to analyze the effects of sex and rat strain. All data analyzed were of normal distribution according to the Shapiro-Wilk test. Tukey´s *post-hoc* test was used for variables showing evidence of interaction between sex and strain. This test was adjusted for multiple comparisons to determine whether sex and strain would have a significant influence on metabolic and inflammatory parameters. Statistical significance was determined at a value of p<0.05. All results are expressed as mean ± SEM.

## 3 Results

### 3.1 Basal metabolic characteristics, body weight and adiposity

**Figure 1:**
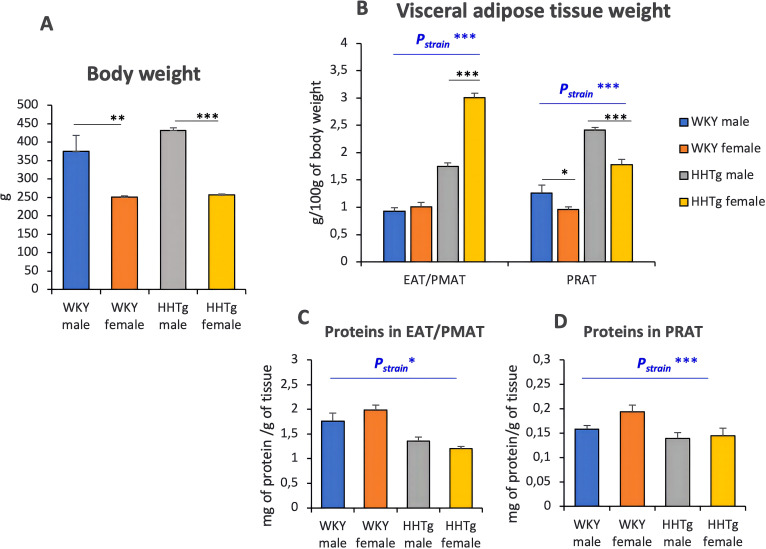
Body **(A)** and visceral adipose tissue weight **(B)**, protein contain in EAT/PMAT **(C)** and PRAT **(D)**. Data are expressed as mean ± SEM and analyzed by two-way ANOVA (P_strain_) and by the Tukey *post hoc* test (multiple comparison between males and females). WKY, Wistar Kyoto rats; HHTg, Hereditary Hypertriglyceridemic rats; EAT, epididymal adipose tissue; PMAT, perimetrial adipose tissue; PRAT, perirenal adipose tissue n=8 per group, * denotes p<0.05, ** denotes p<0.01, *** denotes p<0.001.

As shown in **Figure 1**, the HHTg rat strain exhibited an increased relative weight of visceral adipose tissue compared to age-matched WKY controls; however, there was no difference in whole-body weight between the two strains. While the HHTg rat strain showed increased visceral adiposity, significant sex differences were observed in individual fat depots. Compared to HHTg males, HHTg females exhibited a significant increase in perimetrial adipose tissue (PMAT) and a decrease in perirenal adipose tissue (PRAT), while the opposite was true for HHTg males. The increased visceral adipose tissue in the HHTg strain was accompanied by decreased protein content in both sexes indicating the presence of larger and less metabolically active adipocytes (**Figure 1**).

As shown in **Table 1**, the HHTg rat strain exhibited severe dyslipidaemia characterised by genetically determined hypertriglyceridaemia and decreased HDL cholesterol (**Table 1**). Compared to HHTg males, HHTg females exhibited more severe hypertriglyceridaemia. Conversely, the HHTg rat strain exhibited reduced serum cholesterol levels. Additionally, the HHTg rats strain exhibited hyperuricaemia and the changes in sex hormones. Compared to WKY control females, HHTg females exhibited higher levels of estradiol measured in a postprandial condition, but lower levels of postprandial testosterone. Interestingly, HHTg males exhibited increased serum levels of both estradiol and testosterone compared to WKY control males (**Table 1**).

**Table 1:** Basal metabolic characteristics. .

	WKY male	WKY female	HHTg male	HHTg female	P_STRAIN_	P_SEX_	P_INT_
Serum TAG *(mmol/l)*	0.63 ± 0.02	0.67 ± 0.08	4.35 ± 0.30	5.20 ± 0.39**	<0.001	<0.05	<0.05
Serum cholesterol *(mmol/l)*	2.34 ± 0.04	2.82 ± 0.09***	1.72 ± 0.03	1.46 ± 0.07*	<0.001	n.s.	<0.001
HDL-cholesterol *(mmol/l)*	0.74 ± 0.10	0.82 ± 0.03***	0.57 ± 0.01	0.45 ± 0.02**	<0.001	n.s.	<0.001
Uric acid *(mmol/l)*	33.10 ± 3.26	47.30 ± 3.52	70.25 ± 5.47	76.80 ± 19.32	<0.001	n.s.	n.s.
HOMA-IR	2.68 ± 0.11	2.80 ± 0.10	3.08 ± 0.04	3.05 ± 0.12	<0.01	n.s.	n.s.
Estradiol *(pg/ml)*	13.22 ± 1.35	56.73 ± 6.14	40.06 ± 7.14	102.96 ± 17.96**	<0.01	<0.001	n.s.
Testosterone *(ng/ml)*	3.66 ± 0.34	0.76 ± 0.06***	5.08 ± 0.66	0.58 ± 0.12***	n.s.	<0.001	<0.05

Values are given as mean ± SEM; n=8 for each group; Ps probability reflecting the effect of the strain and were analyzed by two-way ANOVA and by the Tukey *post hoc* test. Two-way ANOVA results: Pstrain denotes the significance of WKY vs, HHTg (strain effect); Psex denotes the significance (sex effect); Pint denotes of sex in both strains (strain vs. sex interaction). For multiple comparison the LSD Fisher´s *post hoc* test was used. *p<0.05; **p<0.01; ***p<0.001. WKY – Wistar Kyoto rats; HHTg – Hereditary Hypertriglyceridemic rats; HOMA-IR homeostatic model assessment for insulin resistance.

### 3.2 Glucose tolerance and insulin sensitivity markers

**Figure 2:**
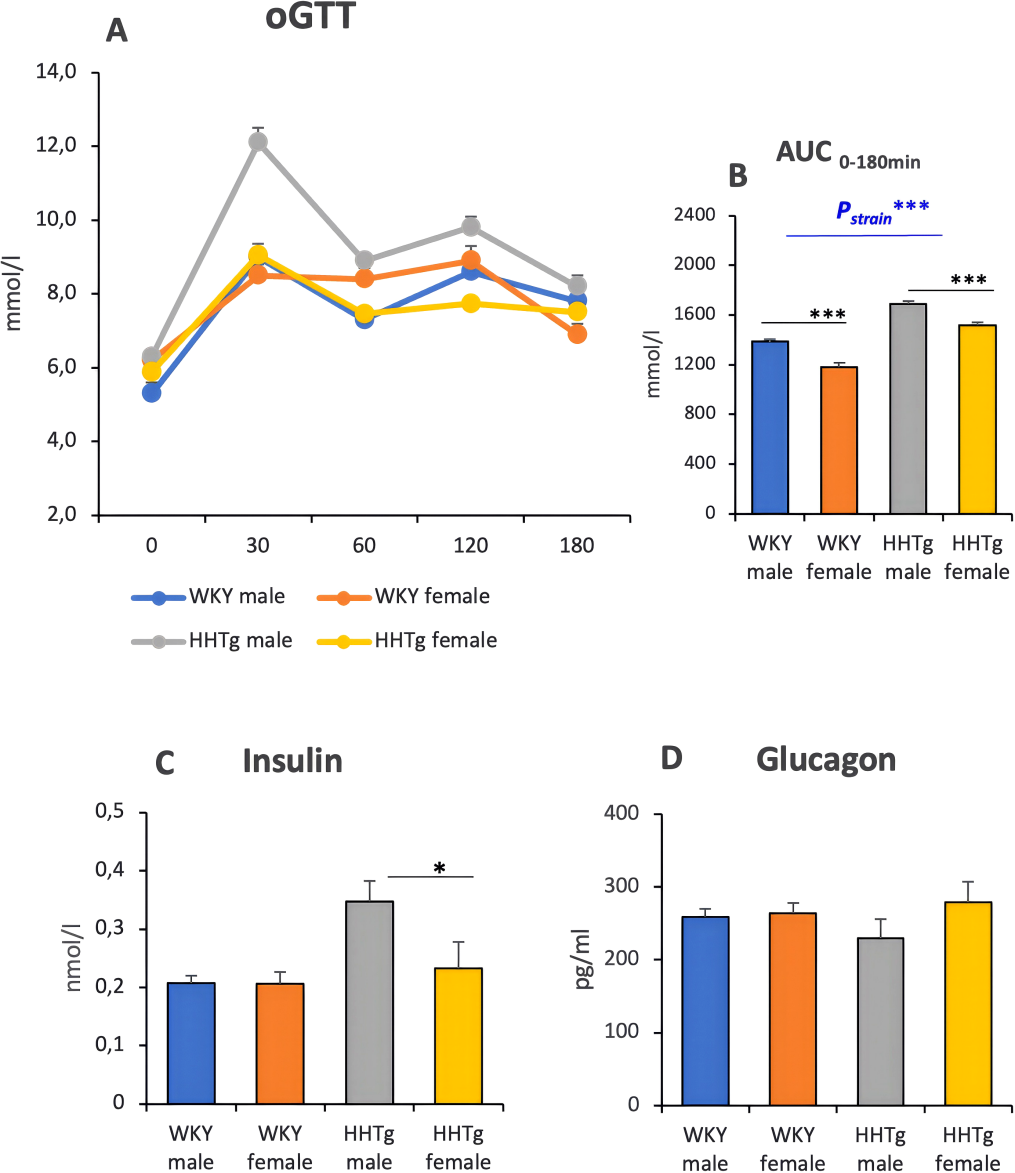
oGTT **(A)** with AUC **(B)**, insulin **(C)** and glucagon **(D)**. Data are expressed as mean ± SEM and analyzed by two-way ANOVA (P_strain_) and by the Tukey *post hoc* test (multiple comparison between males and females). WKY, Wistar Kyoto rats; HHTg, Hereditary Hypertriglyceridemic rats; AUC, area under the curve n=8 per group, * denotes p<0.05, *** denotes p<0.001.

According to the oGTT (see **Figure 2**), the HHTg rat strain exhibited impaired glucose tolerance. Compared to WKY control males, fasting glucose and serum glucose in 30, 60 and 120 minutes during oGTT were significantly higher in HHTg males (p<0.001). On the other hand, females exhibited decreased glucose compared to males in both rat strains. The HHTg rat strain showed hyperinsulinaemia with significantly higher levels in HHTg males than in HHTg females, but no differences were observed in serum glucagon levels (**Figure 2**).

**Figure 3:**
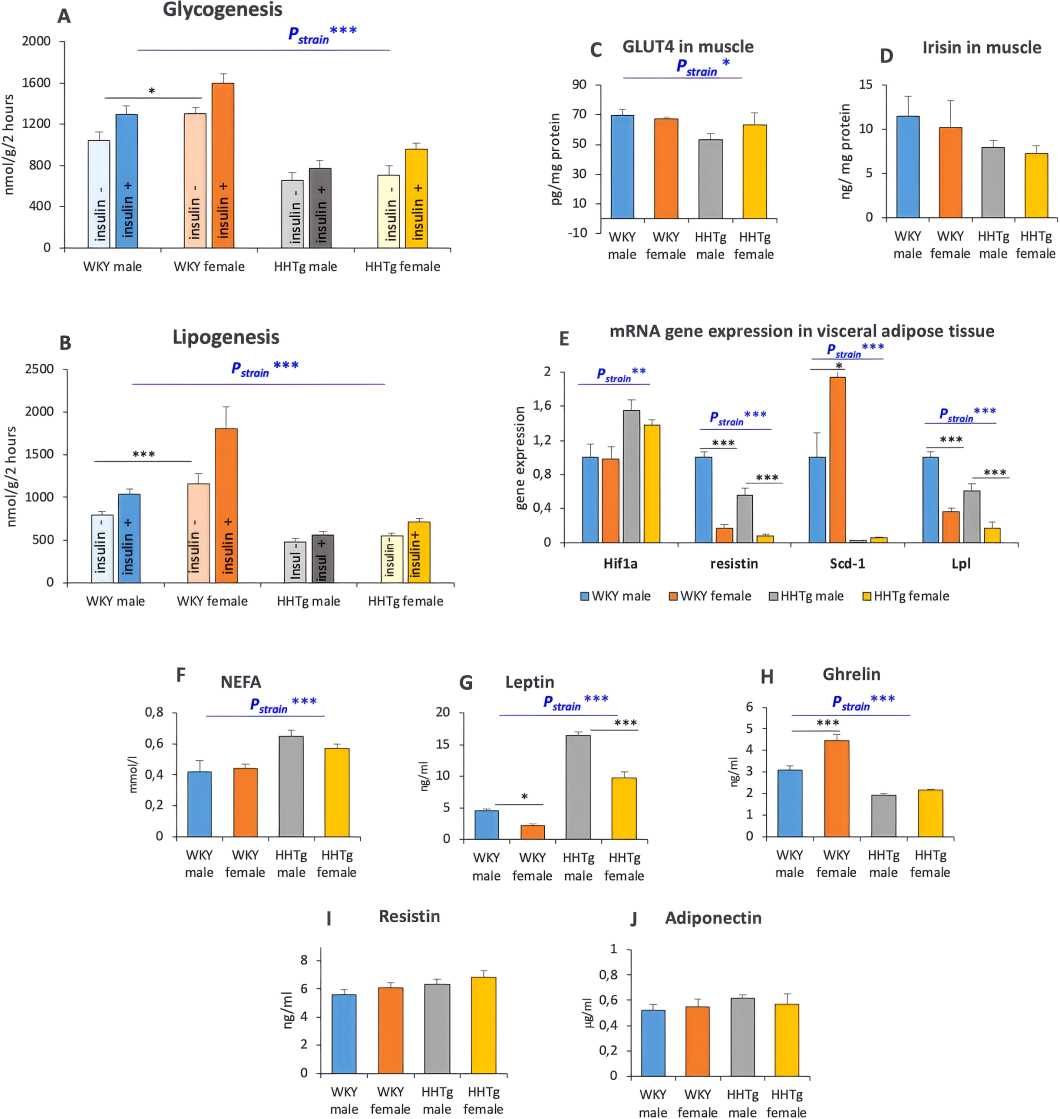
Markers of insulin sensitivity in skeletal muscle **(A)** glycogenesis, **(C, D)** and visceral adipose tissue **(B)** lipogenesis, gene expression in visceral adipose tissue **(E)**, NEFA **(F)** and adipocytokines **(G–J)**. Data are expressed as mean ± SEM and analyzed by two-way ANOVA (P_strain_) and by Tukey *post hoc* test (multiple comparison between males and females). WKY, Wistar Kyoto rats; HHTg, Hereditary Hypertriglyceridemic rats; GLUT4, glucose transporter type 4; NEFA, non-esterified fatty acidn=8 per group, * denotes p<0.05, ** denotes p<0.01, *** denotes p<0.001.

Furthermore, the HHTg strain exhibited decreased insulin sensitivity parameters in both skeletal muscle and visceral adipose tissue (**Figure 3**). In the WKY control group, the females showed significantly increased insulin sensitivity parameters in both tissues compared to males; however, these effects were not significant in the HHTg strain. Impaired insulin sensitivity in the skeletal muscle of HHTg rats was associated with reduced GLUT4 levels in the skeletal muscle but not with significant differences in skeletal muscle irisin levels. In visceral adipose tissue, the relative mRNA gene expression of *HIF1α* was elevated, while the relative *mRNA* gene expression of resistin, *Scd1* and *Lpl* was significantly reduced in HHTg animals compared to controls. This can be associated with lower metabolic activity and higher levels of hypoxia in visceral adipose tissue. Chronically increased NEFA and an imbalance in adipocytokines secretion in HHTg rat strain contribute to insulin resistance in the HHTg rat strain (**Figure 3**). HHTg rats had increased leptin levels, with the increase being more pronounced in males than in females. Total ghrelin serum levels were significantly reduced. There were no significant differences in adiponectin or resistin levels between the rat strains or sexes.

### 3.3 Ectopic lipid and lipotoxic intermediates deposition, genes involved in hepatic lipid metabolism

**Figure 4:**
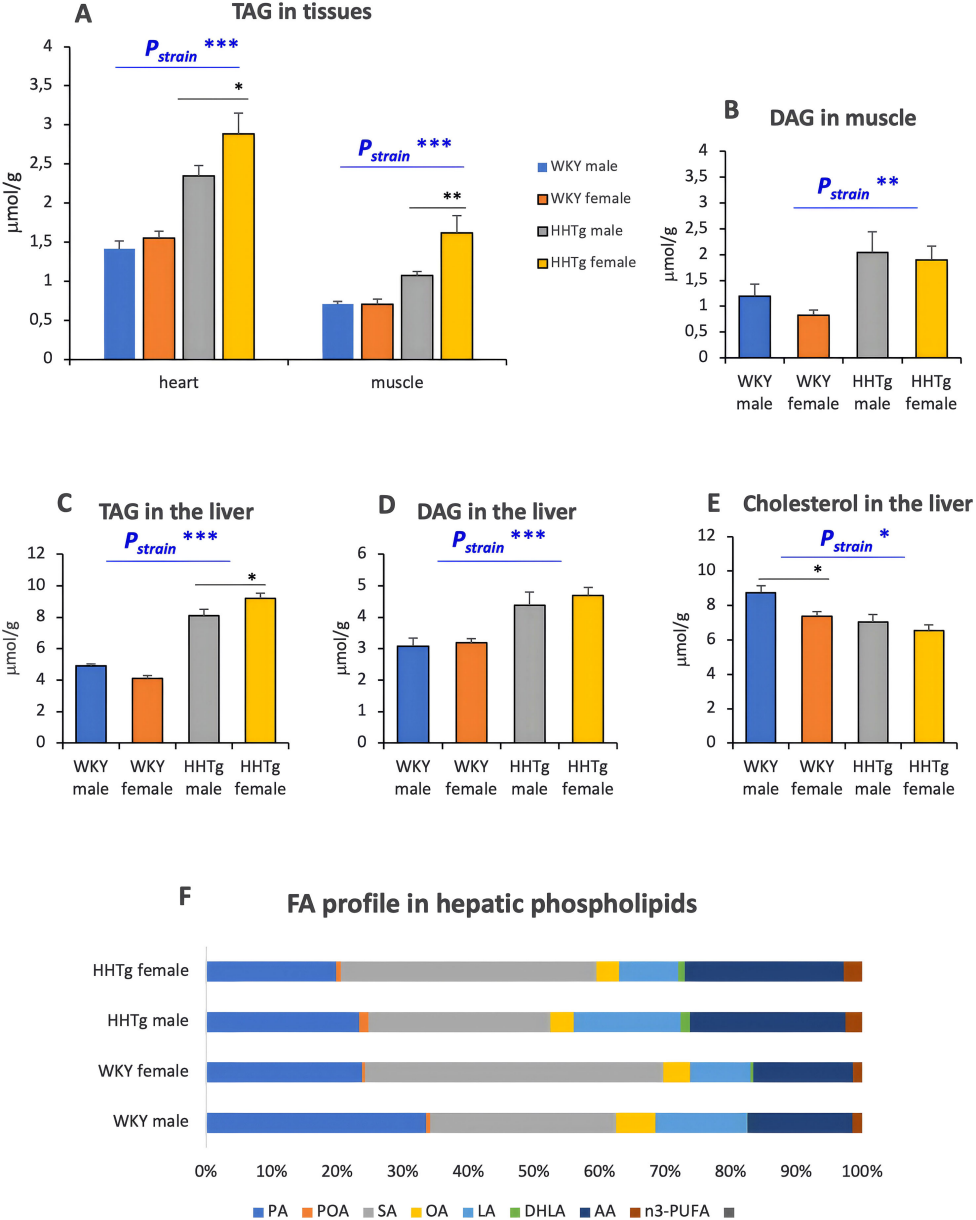
Ectopic lipids **(A, C, E)** and lipotoxic intermediate **(B, D)** accumulation and fatty acid profile in hepatic phospholipids **(F)**. Data are expressed as mean ± SEM and analyzed by two-way ANOVA (P_strain_) and by the Tukey *post hoc* test (multiple comparison between males and females). WKY, Wistar Kyoto rats; HHTg, Hereditary Hypertriglyceridemic rats; TAG, triacylglyceroles; DAG, diacylglyceroles; FA, fatty acid; PA, palmitic acid; POA, palmitoleic acid; SA, stearic acid; LA, linoleic acid; DHLA, dihomo-γ-linoleic acid; AA, arachidonic acid; n=8 per group, * denotes p<0.05, ** denotes p<0.01, *** denotes p<0.001.

Compared to WKY controls, the HHTg rat strain exhibited ectopic TAG accumulation in the liver, heart and skeletal muscle. This accumulation was significantly higher in HHTg females (**Figure 4**). HHTg females showed significantly higher hepatic, heart and skeletal muscle TAG accumulation than age-matched HHTg males. However, there was no differences in lipotoxic DAG accumulation in these tissues between HHTg females and HHTg males. On the other hand, HHTg rat strain exhibited decreased hepatic cholesterol concentration. A significantly higher HOMA-IR index was observed in the HHTg rat strain compared to the control group, which may be associated with IR in the liver (**Table 1**). Changes in the fatty acid profile of hepatic phospholipids (**Figure 4**), particularly the significant increase in the proportion of palmitoleic acid (POA) and dihomo-γ-linoleic acid (DHLA), may contribute to the development of hepatic IR in the HHTg rat strain. On the other hand, the higher arachidonic acid (AA) profile in the HHTg rat strain may rather be related to a protective mechanism against the formation of lipotoxic metabolites derived from arachidonic fatty acid. Sex-specific differences in the hepatic phospholipid fatty acid profile were also observed: compared to HHTg males HHTg females showed decreased POA and DHLA profiles, while saturated fatty acid profile increased. The lower n6-PUFA profile in HHTg females, particularly linoleic acid (LA) can contribute to oxidative stress in the liver.

**Figure 5:**
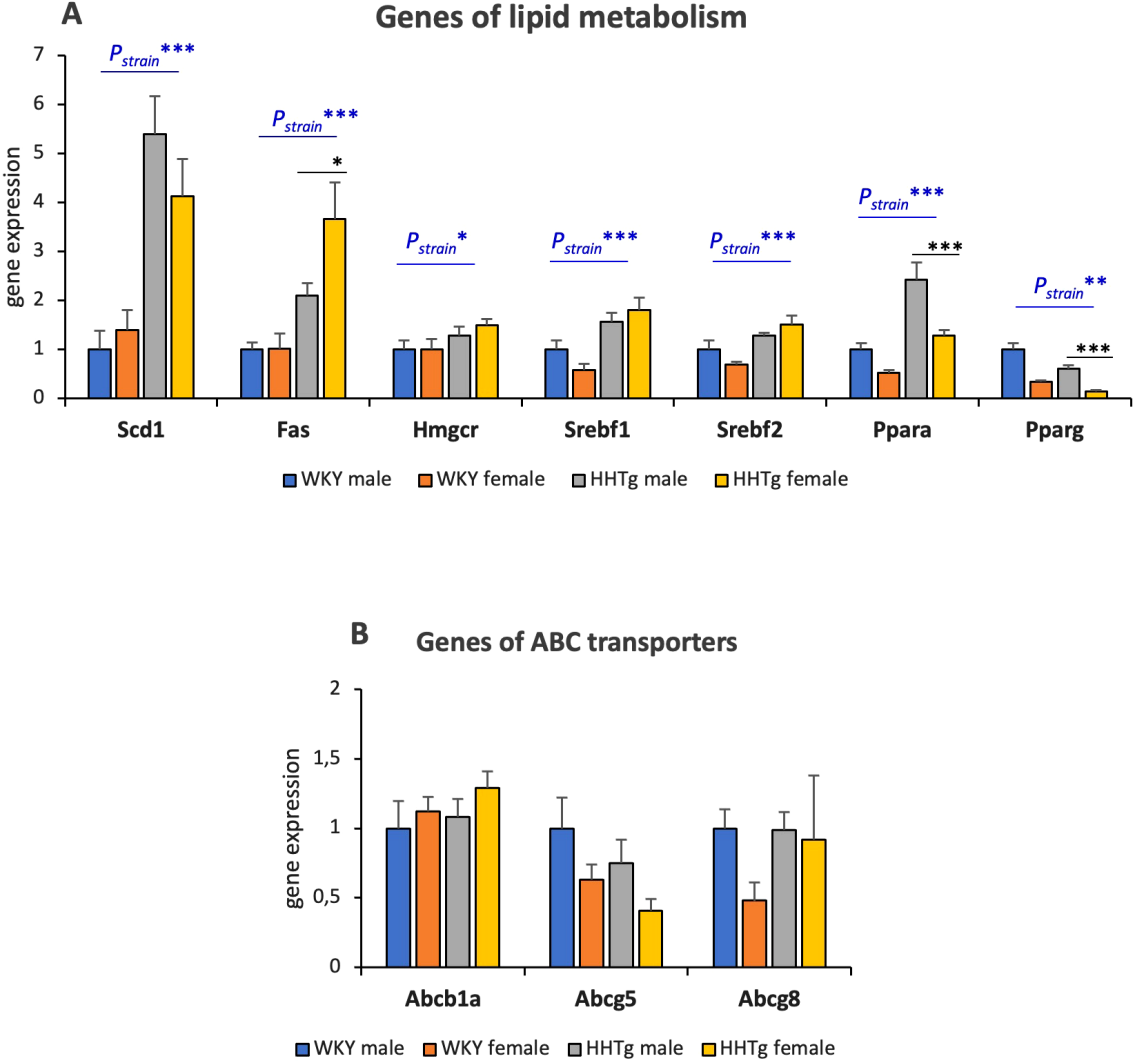
Genes of enzymes, transcription factors **(A)** and ABC transporters **(B)** involved in lipid metabolism in the liver. Data are expressed as mean ± SEM and analyzed by two-way ANOVA (P_strain_) and by the Tukey *post hoc* test (multiple comparison between males and females). WKY, Wistar Kyoto rats; HHTg, Hereditary Hypertriglyceridemic rats; Scd1, stearoyl-Coa desaturase; Fas, fatty acid synthase; Hmgcr, 3-hydroxy-3-methylglutaryl-CoA reductase; Srebf, sterol regulatory element-binding protein; Ppara, peroxisome proliferator-activated receptor alpha; Pparg, peroxisome proliferator-activated receptor gamma n=8 per group, * denotes p<0.05, ** denotes p<0.01, *** denotes p<0.001.

As shown in **Figure 5**, severe dyslipidaemia and ectopic TAG deposition in the HHTg rat strain were associated with significant changes in the expression of genes encoding enzymes and transcription factors involved in liver lipid metabolism. The hepatic mRNA gene expressions of the lipogenic enzymes *Scd1* and *Fas*, as well as of the transcription factors *Srebf1*, *Srebf2* and *Pparα* were markedly increased in *HHTg* rats. In contrast, the *hepatic mRNA* expression of *Pparγ* was significantly decreased in HHTg animals. Additionally, the hepatic mRNA gene expression of *Pparα* and *Pparγ* was significantly lower in HHTg females than in HHTg males. Although there was a slight increase in hepatic mRNA gene expression of *Hmgcr*, no significant differences were observed in the relative gene expression of ABC *transporters* between strains or sexes (**Figure 5**).

### 3.4 Inflammation and oxidative stress in the liver

**Figure 6:**
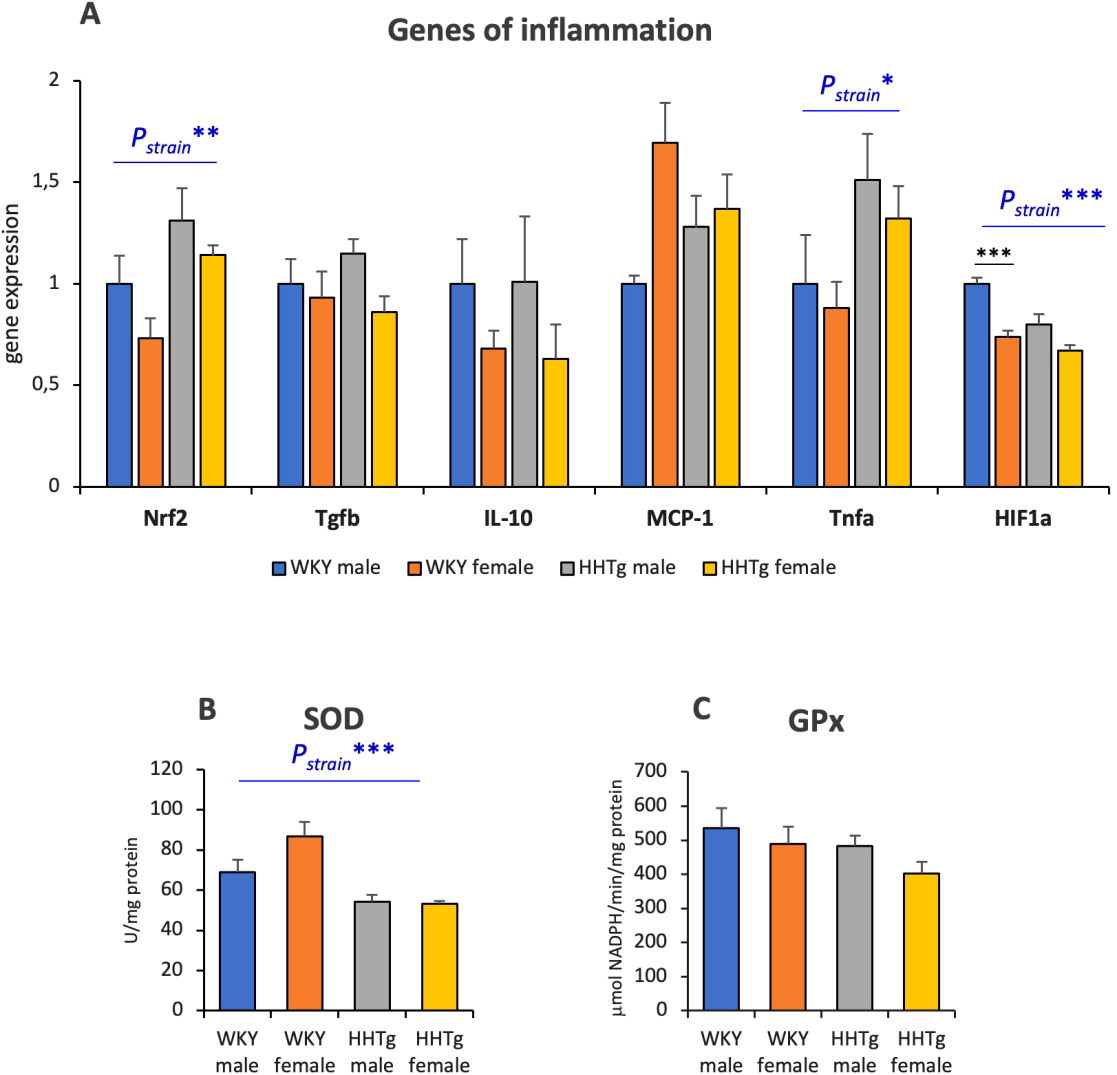
Genes of inflammation **(A)** and activity of antioxidant enzymes SOD **(B)** and GPx **(C)** in the liver. Data are expressed as mean ± SEM and analyzed by two-way ANOVA (P_strain_) and by the Tukey *post hoc* test (multiple comparison between males and females). WKY, Wistar Kyoto rats; HHTg, Hereditary Hypertriglyceridemic rats; Nrf2, nuclear factor erythroid 2-related factor; Tgfβ, transforming growth factor beta; MCP-1, monocyte chemoattractant protein-1; Tnfα, tumor necrosis factor alpha; Hif1α, hypoxia-inducible factor 1 alpha; SOD, superoxide dismutase; GPx, glutathione peroxidase n=8 per group, * denotes p<0.05, ** denotes p<0.01, *** denotes p<0.001.

As shown in **Figure 6**, the relative gene expression of the transcription factor Nrf2 and the pro-inflammatory cytokine Tnfα was higher in the livers of HHTg rats compared to age-matched controls, while the hepatic mRNA gene expression of Hif1α was lower, with this effect being more pronounced in females. Decreased antioxidant enzyme SOD activity in the liver can contribute to hepatic oxidative stress in the HHTg rat strain. On the other hand, there were no changes in GPx activity in the liver (**Figure 6**).

## 4 Discussion

The major findings of this study include sex differences in metabolic disorders associated with prediabetes, as well as sex differences in susceptibility to the development of prediabetic comorbidities that are not accompanied by excessive body weight or induced by dietary intervention. Human studies indicate that obesity is a stronger risk factor for men than for women (18). Furthermore, our study reveals that males are at a higher risk of developing metabolic damage than females of reproductive age, even when obesity is not a factor, with IR and NAFLD playing a particularly significant role.

The prediabetic rat strain used in this study was not obese in terms of body weight; however visceral adiposity was observed particularly in females. Additionally, sex-specific differences in the distribution of visceral adipose tissue were observed. Prediabetic females had a higher weight of perimetrial adipose tissue, whereas prediabetic males had a higher weight of perirenal adipose tissue. The increased visceral adiposity observed in this prediabetic strain may be partly exacerbated by the significantly higher levels of estrogen present in both sexes, which contribute to lipid accumulation. In addition to the amount of adipose tissue, adipocyte size and metabolic activity also matter. A decrease in protein concentration in prediabetic animals indicates larger, less metabolically active adipocytes. Differences in the amount and distribution of fat between the sexes, particularly with regard to certain types of visceral adipose tissue, may contribute to sex-specific differences in metabolic disorders such as IR, NAFLD, low-grade inflammation, and differences in adipocytokine secretion (19).

Sex hormones play a key role in determining insulin sensitivity as well as of glucose and lipid metabolism. They may therefore play a role in the development of prediabetic metabolic disorders in a sex-specific manner. Interestingly, the HHTg strain exhibited increased estradiol levels in both females and males, which can lead to ectopic lipid deposition. Conversely, testosterone levels increased in prediabetic males and decreased in prediabetic females, which may have rather favorable metabolic effects. Testosterone appears to have beneficial metabolic effects in men and detrimental effects in women. Decreased testosterone levels are an important sex-specific risk factor for the development of diabetes in men (20). However, several studies involving peri- or postmenopausal women have demonstrated a significant correlation between increased testosterone levels and impaired glucose homeostasis, IR and an increased risk of diabetes (21). In addition to sex hormones, other factors such as inflammation or metabolic activity of adipose tissue may contribute significantly to sex-based differences in the development of metabolic disorders.

In this study, the prediabetic strain exhibited impaired glucose tolerance and IR which was more pronounced in prediabetic males. Compared to prediabetic females, prediabetic males showed a worse oGTT, higher insulinemia and NEFA levels, which may contribute to sex differences in diabetes onset. Impaired glucose tolerance is the primary manifestation of prediabetes in males, leading to an earlier onset of diabetes. In the postprandial state, men have higher plasma NEFA levels than women due to increased lipolysis, which can be related to the anti-lipolytic action of insulin (22, 23). In line with previous studies on rodents, diet-induced insulin resistance female mice showed greater suppression of lipolysis in response to insulin than male mice (24).

However, the key metabolic abnormality associated with prediabetes and preceding the onset of diabetes is IR itself. In this study, the prediabetic strain exhibited impaired insulin sensitivity in peripheral tissues and in the liver compared to the control group. While no significant sex differences in insulin sensitivity parameters were found between prediabetic males and females in this study, prediabetic females showed a clear trend towards better insulin sensitivity in skeletal muscle. These results are consistent with those of human studies, which generally demonstrate that women exhibit greater systemic insulin sensitivity and glucose tolerance than men despite having higher body fat mass (18).

IR first appears in adipose tissue and in the liver (19), and various triggers on insulin signaling defects are tissue-dependent. In adipose tissue, inflammation and hypoxia play a key role in IR development (25, 26), while in skeletal muscle lipotoxicity seems to be the predominant mechanism of IR development (27). Consistent with our results, IR in prediabetic rats was associated with hypoxia, low-grade chronic inflammation and an imbalance in adipocytokine secretion in visceral adipose tissue, with sex-related differences observed. Increased gene expression of Hif1α can contribute to higher hypoxia and chronic inflammation in visceral adipose tissue. According to these results and our previous findings (13), HHTg males exhibited higher leptin levels, whereas females exhibited higher levels of IL-6. The higher leptin levels found in prediabetic males may contribute to both insulin and leptin resistance, although there is no direct correlation with visceral adipose tissue mass. While higher leptin levels are usually found in women surprisingly our study found higher leptin levels in HHTg males than in HHTg females (13). However, no changes were observed in resistin or adiponectin. Generally, visceral adiposity is strongly linked to IR, but higher visceral adiposity in HHTg females was not associated with worsening IR in visceral adipose tissue. In addition, sex-specific differences in Scd1 and Lpl gene expression which are involved in adipose tissue lipid metabolism can contribute to IR in visceral adipose tissue and to sex differences in body fat distribution. According to a multi-omics integrative study involving approximately 100 strains of mice (28), sex hormones play a role in regulating processes in the liver and adipose tissue. However, testosterone has a more significant impact on both tissues, while estrogen is more effective in adipose tissue. Consistent with human studies, the risk of IR and type 2 diabetes is lower in premenopausal obese women than in obese men, despite their higher adiposity (29). IR is strongly correlated with NAFLD mainly in males. Differences in insulin sensitivity may partially account for the sex-related differences in NAFLD. Moreover, animal studies involving mice have shown that a high-fat diet leads to more severe conditions associated with NAFLD in male mice than in female mice (9, 10).

In this study, IR in skeletal muscle in prediabetic animals was accompanied by ectopic lipid deposition and decreased GLUT4 concentration. Although prediabetic females exhibited higher muscle TAG deposition than prediabetic males, no differences were observed in the concentration of lipotoxic DAG in muscles. It has recently been suggested that there is an inverse relationship between intramuscular TAG content and whole-body insulin sensitivity (30). However, this is not a causal relationship. Intramuscular lipids are inversely associated with insulin sensitivity due to the accumulation of lipotoxic intermediates (31) which impair insulin signaling. Our results are consistent with the study of Hoeg (32), which found that higher intramuscular TAG in women does not impair insulin sensitivity and signaling.

Lipotoxic intermediates or specific lipids such as DAG, ceramides or phospholipids and lysophospholipids are involved in IR development and may contribute to sex differences in IR development (4). According to our previous lipidomic results (33), IR in the skeletal muscle in prediabetic males is driven by the accumulation of lipotoxic DAG and ceramides, alongside a reduction in specific phospholipids and lysophospholipids. Furthermore, the observed lipid alterations are consistent with impaired fatty acid oxidation in muscle tissue. Interestingly, differences in non-oxidative glucose disposal may contribute to sex differences in insulin sensitivity. Despite having lower lean mass, women have been shown to exhibit higher insulin-stimulated glucose uptake in skeletal muscle than men (34). In addition, sex differences exist in skeletal muscle substrate metabolism. Men have been found to have higher glycogen storage and oxidative capacity, while women have been found to have higher lipid uptake and storage (35). Therefore, females are better able than males to utilize lipids via fatty acid oxidation, thereby reducing the accumulation of lipotoxic intermediates. However, other mechanisms may be involved. Sex differences in insulin sensitivity may be due to differences in the insulin signaling cascade or GLUT4 translocation (36).

IR manifestation also coexists with hyperuricaemia. Although the pathogenesis is not fully understood, it may play a role in IR development. Higher levels of uric acid have been strongly associated with an increased prevalence of prediabetes (37), and this association has been found to be more significant in men. Consistent with this finding, the prediabetic strain exhibited hyperuricaemia. The increase was 112 per cent in males compared to 62 per cent in females. Hyperinsulinaemia promotes urate accumulation through enhanced reabsorption and impaired renal urate secretion. Elevated uric acid has been shown to impair pancreatic β-cell function by inducing oxidative stress, diminishing glucose-stimulated insulin secretion, and promoting apoptosis (38). Thus, elevated uric acid exacerbates IR, partly via oxidative stress and inflammatory cytokine release.

The increased ectopic TAG accumulation in females compared to males may be due to estrogens, as estrogen increases the expression of genes involved in de novo lipogenesis (39). In addition, estrogens increase genes involved in fatty acid oxidation which contribute to reducing lipotoxic DAG accumulation. As the Schiffrin study points out, it is also possible that each sex prefers different forms of lipid storage (40). In a mouse model of hepatic steatosis, lipidomics analysis revealed hepatic accumulation of short and highly saturated triacylglyceroles in females, while males showed an enrichment of long and unsaturated hydrocarbon chains. These results are consistent with our findings and can partly explain why less DAG is produced in females despite higher TAG levels in the liver (33). Increased gene expression of the main lipogenic enzymes Scd1 and Fas increases de novo lipogenesis and can participate to hepatic lipid accumulation in the HHTg strain. Markedly increased hepatic gene expression of Srebf1, a target gene of lipid synthesis and a key regulator of hepatic lipogenesis, may also play a role. In addition, high level of insulin activates Srebf1 production, facilitating fatty acids storage as triacylglyceroles and leading to hepatic steatosis (41). Thus, Srebf1 acts as the master regulator of hepatic steatosis, and, together with decreased Pparγ gene expression, it can contribute to hepatic IR. Other key regulators responsible for controlling genes involved in lipid homeostasis and inflammation are the Nrf2 family members. Furthermore, Nrf2 nuclear receptors affect the gene expression of Srebf1 and Pparγ (42). Decreased gene expression of Pparγ in the liver can contribute to hepatic IR and inflammation. In the liver, Pparγ exhibits an anti-inflammatory effect by inhibiting the production of pro-inflammatory cytokines and pro-inflammatory immune cells (43). Therefore, decreased hepatic Pparγ may contribute to chronic inflammation in the liver. Furthermore, Pparγ is involved in the regulation of glucose homeostasis and can therefore affect insulin sensitivity in the liver.

Taken together, the changes in liver gene expression support hepatic lipid accumulation primarily through increased lipogenesis, and to a lesser extent, increased fatty acid synthesis. Although sex differences in hepatic gene expression are not very pronounced in the HHTg strain, sex-dimorphic hepatic gene expression differs in the prediabetic strain compared to the controls. Notably, the sex-dimorphic alterations in Scd1, Fas and Srebf differ in the prediabetic strain, indicating that hepatic steatosis can alter sex-dimorphic gene expression. Conversely, increased hepatic Fas expression corresponds to more pronounced triacylglyceroles accumulation in prediabetic females than in males.

The fatty acid profile of hepatic membrane phospholipids can affect membrane fluidity as well as various signaling pathways including those involved in insulin and inflammatory signalization (44). According to our results, changes of the fatty acid profile in hepatic phospholipids in prediabetic animals indicate hepatic IR development, oxidative stress and inflammation. Compared to females, HHTg males exhibit more pronounced changes related to IR and oxidative stress, indicating that steatosis develops more significantly in males. Oxidative stress and inflammation are supported by a higher proportion of AA and LA, which is partially offset by a higher proportion of n3-PUFA. In addition, increased Scd1 gene expression contributes to higher POA profile, which is strongly associated with the IR condition.

In this study, increased Tnfα expression together with reduced SOD activity further aggravated hepatic inflammation and oxidative stress in prediabetic strain. Changes in circulating pro-inflammatory factors are related to IR in adipose tissue, which is manifested by an imbalance in adipocytokine secretion. Circulating pro-inflammatory cytokines contribute to chronic hepatic inflammation, and consequently to the NAFLD development (45). These results are consistent with findings showing that leptin and the pro-inflammatory TNFα are strongly correlated with fatty liver disease and IR in adipose tissue. Furthermore, the results are consistent with a human study, which suggests that the severity of fatty liver disease is associated with adipose tissue dysfunction and IR rather than BMI (46). Results indicate that not only the development but also the consequence of NAFLD might be sex-dimorphic.

## 5 Conclusion

Sex-specific differences may include variations in fat distribution, lipid metabolism and storage, and inflammatory manifestations. In our study, severe dyslipidaemia and subsequent ectopic lipid accumulation present in the Hereditary Hypertriglyceridaemic - HHTg rat strain were associated with insulin resistance, glucose intolerance and low-grade chronic inflammation. Despite exhibiting more pronounced dyslipidaemia, ectopic lipid accumulation and visceral adiposity, prediabetic female HHTg rats demonstrated better glucose tolerance and some of insulin sensitivity markers than male HHTg rats. Our experimental data indicate that the onset of prediabetes and its early pathophysiological demonstrations, such as insulin resistance and fatty liver, are more prevalent in males than in females of reproductive age, regardless of the presence of obesity. Our animal study focuses on sex-specific differences related to prediabetic tissue damage that are independent of obesity and which could represent the therapeutic targets.

## Data Availability

The original contributions presented in the study are included in the article/supplementary material, further inquiries can be directed to the corresponding author/s.
